# Supplementary material for: Dendrogenin A drives LXR to trigger lethal autophagy in cancers
Source: Nat Commun. 2017 Dec 4;8:1903. doi: 10.1038/s41467-017-01948-9 (PMC5712521; doi:10.1038/s41467-017-01948-9)
Supplement: Supplementary file 1 — Supplementary Information [file 41467_2017_1948_MOESM1_ESM.pdf]

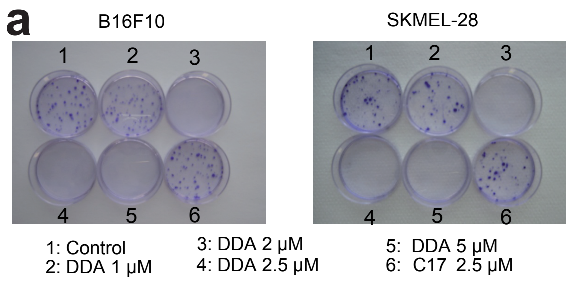

b

Cell line	BRaf mutation	Cytotox EC_{50} μM	Clono IC_{50} μM
SKMEL-28 (*H*)	V600E	2.1± 0.2	1.4± 0.6
B16F10 (*M*)	WT	0.7± 0.1	1.2± 0.3
A375 (*H*)	V600E	1.8± 0.2	1.5± 0.3
WM35 (*H*)	V600E	1.6± 0.1	1.4± 0.2
WM-266-4 (*H*)	WT	2.7± 0.4	1.2± 0.4
WM115 (*H*)	V600E	2.2± 0.2	1.5± 0.3
SKMEL-2 (*H*)	WT	2.3± 0.1	1.4± 0.4

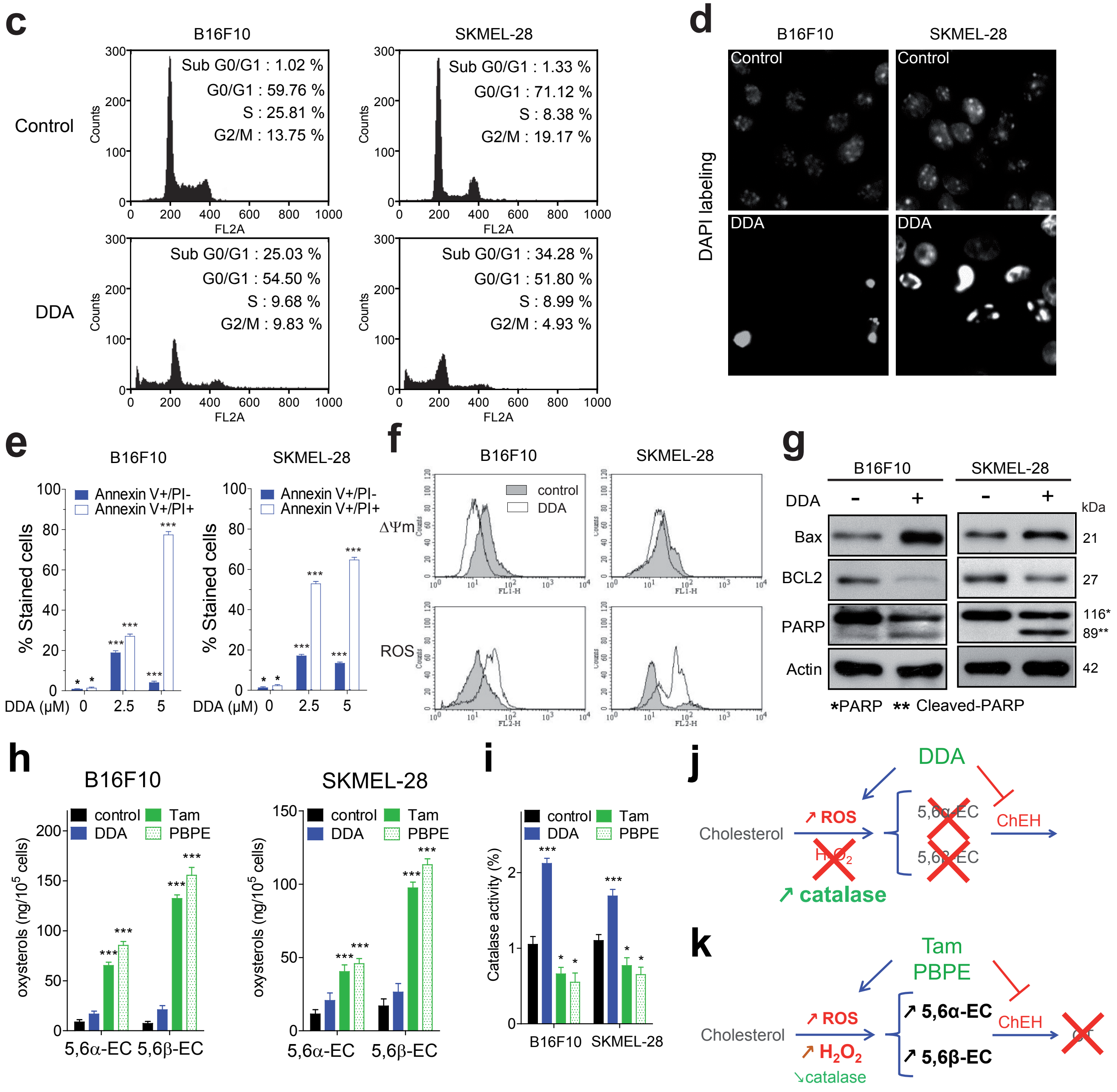

Supplementary Fig. 1. **a** DDA inhibited melanoma colony formation. Melanoma cells were treated with solvent vehicle, DDA or its inactive isomer C17 for 72 h. Plates were stained with a 0.5% solution of crystal violet in water and analyzed by microscopy. Images are representative of 5 independent experiments. **b** Evaluation of DDA-induced cytotoxicity (cytotox) and clonogenicity (clono) inhibition in a subset of human (*H*) and mouse (*M*) melanoma cell lines. The BRaf status in melanoma cells lines is shown. Cells were exposed for 72 h to increasing concentrations of DDA ranging from 0.1 to 10 µM and EC_{50} and IC_{50} were determined. **c** Cell cycle distribution of cancer cells treated with solvent vehicle (control) or 5 µM DDA for 48 h, measured by flow cytometry. **d** DDA induced chromatin condensation. Cells were treated for 24 h with solvent vehicle (control) or 5 µM DDA and stained with DAPI. (e) DDA induced the production of annexin-V-positive and propidium iodide (PI)-negative cells. Bars represent S.E.M. (*P<0.05, *t* test). **f** DDA induced a modification of the mitochondrial membrane potential ($\Delta\psi_m$) and stimulated the production of reactive oxygen species (ROS). **g** Effect of DDA on Bax, Bcl2 and PARP expression. Cells were treated with solvent vehicle (control) or 5 µM DDA for 24 h. Images of blots and microscopy are representative of 3 independent experiments. **h** Quantification of 5,6α-EC and 5,6β-EC in melanoma cells incubated for 48h with solvent vehicle (0.1% EtOH), 2.5 µM DDA, 10 µM Tam or 40µM PBPE. 5,6-EC were quantified by GC/MS. The results are reported as ng 5,6-EC per 10^5 cells. **i** DDA but not ChEH inhibitors Tam and PBPE stimulates catalase activity in melanoma cells. Cells were treated as above and catalase activity was measured as described on the "Methods" section. Data are the means ± S.E.M. of 3 independent experiments performed in triplicate (*P<0.05, **P<0.01, ***P<0.001, *t* test). **j** Scheme describing that DDA induced oxidative stress and catalase activity which destroys H_2O_2 and impaired 5,6-ECs formation and accumulation, despite the inhibition of ChEH. **k** Scheme describing that ChEH inhibitiors Tam and PBPE induce oxidative stress but not catalase activity, which led to 5,6-ECs formation and accumulation.

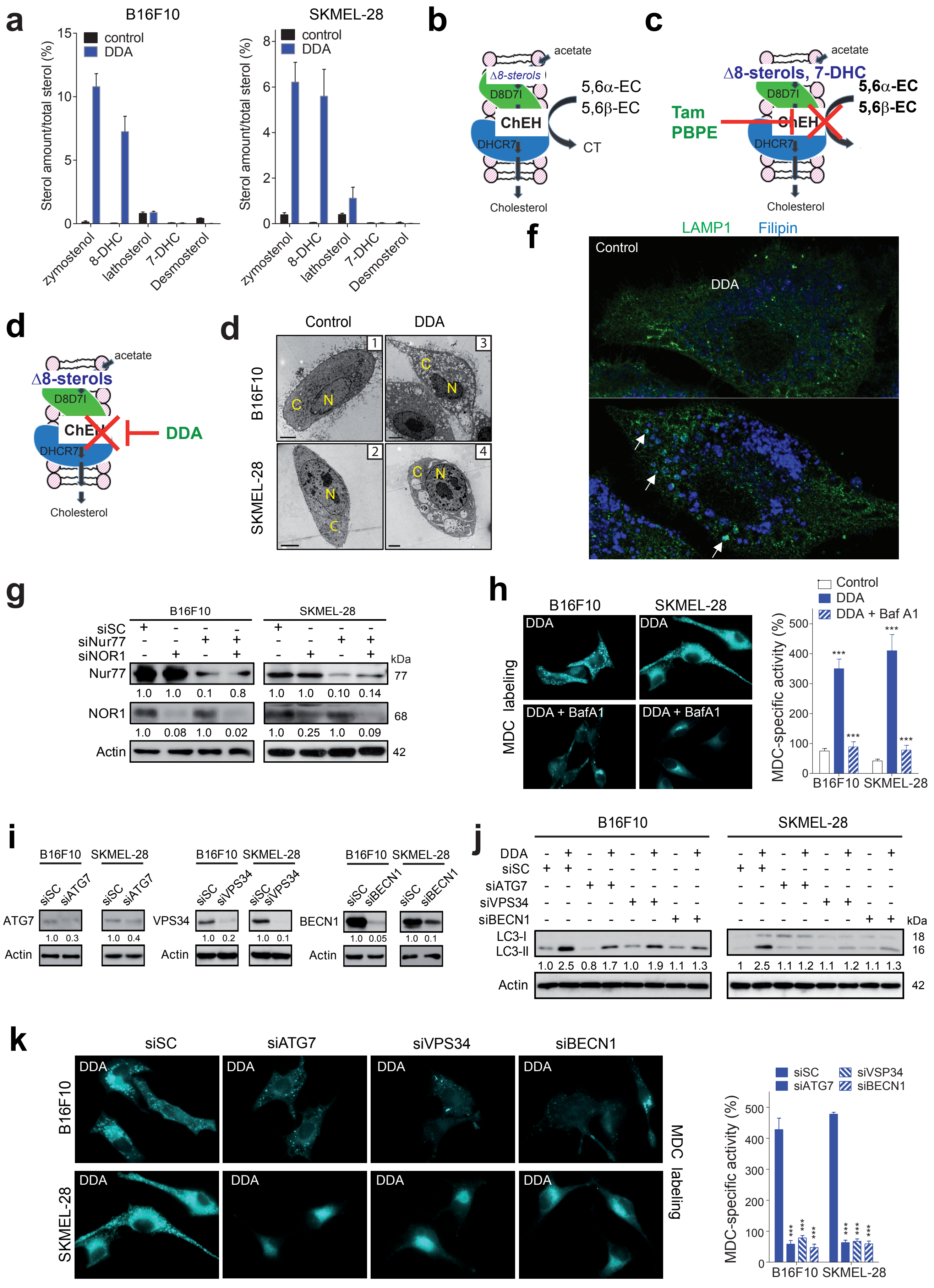
a
B16F10
SKMEL-28
control
DDA
Sterol amount/total sterol (%)
zymostenol
8-DHC
lathosterol
7-DHC
Desmosterol
b
acetate
Δ8-sterols
D8D7I
ChEH
DHCR7
5,6α-EC
5,6β-EC
CT
Cholesterol
c
acetate
Δ8-sterols, 7-DHC
D8D7I
ChEH
DHCR7
5,6α-EC
5,6β-EC
Tam
PBPE
Cholesterol
d
acetate
Δ8-sterols
D8D7I
ChEH
DHCR7
DDA
Cholesterol
d
Control
DDA
B16F10
SKMEL-28
C
N
1
2
3
4
f
LAMP1
Filipin
Control
DDA
g
B16F10
SKMEL-28
siSC
siNur77
siNOR1
kDa
Nur77
77
1.0 1.0 0.1 0.8
1.0 1.0 0.10 0.14
NOR1
68
1.0 0.08 1.0 0.02
1.0 0.25 1.0 0.09
Actin
42
h
B16F10
SKMEL-28
DDA
DDA + BafA1
MDC labeling
Control
DDA
DDA + Baf A1
MDC-specific activity (%)
***
i
B16F10
SKMEL-28
siSC
siATG7
siVPS34
siBECN1
ATG7
VPS34
BECN1
Actin
1.0 0.3
1.0 0.4
1.0 0.2
1.0 0.1
1.0 0.05
1.0 0.1
j
B16F10
SKMEL-28
DDA
siSC
siATG7
siVPS34
siBECN1
kDa
LC3-I
LC3-II
18
16
1.0 2.5 0.8 1.7 1.0 1.9 1.1 1.3
1 2.5 1.1 1.2 1.1 1.2 1.1 1.3
Actin
42
k
siSC
siATG7
siVPS34
siBECN1
B16F10
SKMEL-28
DDA
MDC labeling
siSC
siATG7
siVSP34
siBECN1
MDC-specific activity (%)
***

Supplementary Fig. 2. DDA induced the accumulation of cholesterol precursors in melanoma cells. **a** Quantification of sterol accumulation in cells after 48 h treatment with or without 2.5 μM DDA. DHC: dehydrocholesterol, Data are the means ± S.E.M. of 3 independent experiments performed in triplicate. **b** ChEH catalyzes 5,6α-EC and 5,6β-EC hydration into CT. ChEH is made of D6D7I and DHCR7 which catalyze the isomerization of Δ8-sterols and the reduction of 7-DHC respectively. **c** Tam and PBPE inhibit D8D7I leading to Δ8-sterols accumulation. Tam and PBPE stimulate 5,6-epoxydation of cholesterol and inhibit ChEH, triggering 5,6-ECs accumulation. **d** DDA inhibits D8D7I leading to Δ8-sterols accumulation. DDA did not stimulate 5,6-epoxydation of cholesterol and did not induce 5,6-ECs accumulation. **e** Representative EM images of cells treated for 24 h with solvent vehicle (panels 1-2) or 2.5 μM DDA (panels 3-4). N: nucleus, C: cytoplasm. Bars: 5 μm for panels 1-2, 10 μm for panels 3-4. **f** DDA induced the accumulation of sterol-rich vesicles expressing the lysosomal marker LAMP1. Cells were treated with control solvent vehicle or 2.5 μM DDA for 48 h, then fixed and stained with filipin and anti-LAMP1 antibodies and analyzed by confocal fluorescence microscopy. **g** Representative immunoblots of Nur77 and NOR1 protein expression in cells transfected with scramble siRNA (siSC), Nur77 siRNA (siNur77), NOR1 siRNA (siNOR1) or both (siNur77 + siNOR1). Images of blots and microscopy are representative of 3 independent experiments. **h** The pharmacological inhibitor of autophagy, Bafilomycin A1 (Baf A1), inhibited the accumulation of MDC-labeled vesicles in cells treated with DDA. Cells were treated with solvent vehicle (control) or 2.5 μM DDA for 24 h with or without 1 μM Baf A1, then stained with MDC and observed by fluorescence microscopy. Bars represent S.E.M. (**P<0.01, *t* test). **i** (B) The autophagic proteins ATG7, VPS34 and BECN1 were knocked down by transfection with specific siRNAs. This was confirmed by immunoblotting 96 h after transfection. **j** Immunoblots of LC3 protein expression in cells transfected with siSC, siNur77 and siNOR1. 72 h after transfection, cells were treated for a further 24 h with 2.5 μM DDA. All images and densitometry values are representative of 3 independent experiments. **k** The knockdown of ATG7, VPS34 and BECN1 inhibited the production of MDC-positive vesicles induced by DDA in B16F10 and SKMEL-28 cells. Cells were knocked down by transfection with specific siRNAs, then treated with solvent vehicle (control) or 2.5 μM DDA for 24 h, stained with MDC and observed by fluorescence microscopy. Images of blots and microscopy are representative of 3 independent experiments.

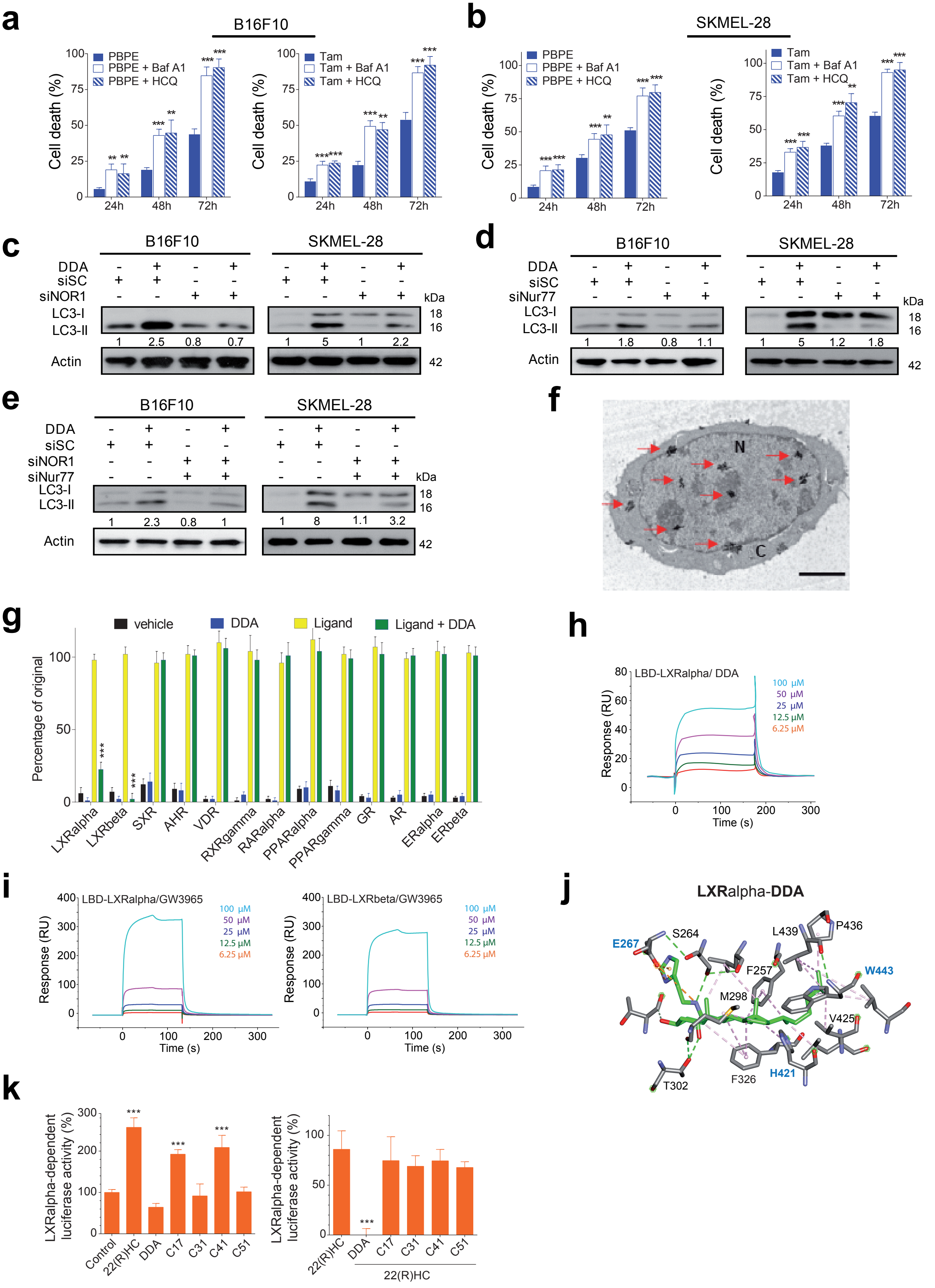
a
B16F10
PBPE
PBPE + Baf A1
PBPE + HCQ
Tam
Tam + Baf A1
Tam + HCQ
Cell death (%)
24h
48h
72h
b
SKMEL-28
c
DDA
siSC
siNOR1
LC3-I
LC3-II
Actin
kDa
18
16
42
1 2.5 0.8 0.7
1 5 1 2.2
d
siNur77
1 1.8 0.8 1.1
1 5 1.2 1.8
e
1 2.3 0.8 1
1 8 1.1 3.2
f
N
C
g
vehicle
DDA
Ligand
Ligand + DDA
Percentage of original
LXRalpha
LXRbeta
SXR
AHR
VDR
RXRgamma
RARalpha
PPARalpha
PPARgamma
GR
AR
ERalpha
ERbeta
h
LBD-LXRalpha/ DDA
100 μM
50 μM
25 μM
12.5 μM
6.25 μM
Response (RU)
Time (s)
i
LBD-LXRalpha/GW3965
LBD-LXRbeta/GW3965
j
LXRalpha-DDA
E267
S264
L439
P436
W443
F257
M298
V425
H421
F326
T302
k
LXRalpha-dependent luciferase activity (%)
Control
22(R)HC
DDA
C17
C31
C41
C51
22(R)HC

Supplementary Fig. 3. Time course study of the cytotoxic activities of two D8D7I inhibitors, PBPE and Tamoxifen (Tam), in the presence of autophagy inhibitors. B16F10 **a** and SKMEL-28 **b** were treated with 2.5 μM DDA, 40 μM PBPE or 10 μM tamoxifen (Tam) for 24, 48 and 72 h in the presence or absence of the autolysosome inhibitors Baf A1 or Hydroxychloroquine (HCQ). **c-e** Immunoblots of LC3 protein expression in cells transfected with siSC, siNur77 and siNOR1. 72 h after transfection, cells were treated for a further 24 h with 2.5 μM DDA. All images and densitometry values are representative of 3 independent experiments. **f** Representative autoradiograph of B16F10 cells incubated with [^{14}C]-DDA for 6 h and analyzed by TEM. N: nucleus, C: cytoplasm. Bar: 5 μm. Red arrows indicate the presence of [^{14}C]-DDA in the nucleus. **g** Effect of DDA on a subset of nuclear receptors. HEK293T cells were transfected with reporter luciferase genes under the transcriptional control of the indicated receptors and their corresponding obligatory partners, and were incubated in the absence or presence of their cognate agonist. Transfected cells were treated or not for 8 h with 2.5 μM DDA and analyzed for luciferase activity. **h** SPR sensorgrams showing the binding of a series of concentrations of DDA on LBD-LXRα, captured on a Biacore sensor chip. Data are representative of 3 independent experiments. **i** SPR sensorgrams showing the binding of a series of concentrations of GW3965 on LBD-LXRα and LBD-LXRβ. Data are representative of 3 independent experiments. **J** Molecular docking simulations highlighting that DDA can be well accommodated within the LBD-LXRα and LBD-LXRβ. Amino acid side chains that interact with DDA are represented (in black). The names of the amino acids known to interact with known LXR ligands are colored in blue. Gray: carbon atoms, white: hydrogen atoms, red: oxygen atoms, blue: nitrogen atoms, yellow: sulfur atoms.**k** Analysis of LXRα-dependent agonistic or antagonistic activities by DDA analogs. Data from a, b, g, k are the means ± S.E.M. of 3 independent experiments performed in triplicate (*P<0.05, **P<0.01, ***P<0.001, *t* test).

a

	B16F10							
	DDA		22(R)HC		GW		TO	
	siCT	siLXRβ	siCT	siLXRβ	siCT	siLXRβ	siCT	siLXRβ
ABCA1	0.4±0.1	1.2±0.1	12.5±2.5	4.2 ±0.9	14.3±1.6	2.6±0.1	8.5±1.8	2.0±0.4
ABCG1	1.4±0.1	0.9±0.1	0.7±0.1	1.1±0.1	2.1±0.4	1.1±0.2	1.1±0.3	1.0±0.1
ABCG5	3.5±0.1	1.0±0.1	4.2±0.2	2.0±0.5	1.9±0.5	0.9±0.1	3.6±0.6	1.0±02
ABCG8	1.6±0.2	1.2±0.1	1.6±0.2	1.0±0.1	1.7±0.2	0.9±0.1	1.5±0.1	1.1±0.2
SULT2b1b	1.6±0.1	0.8±0.1	1.5±0.3	1.1±0.2	1.2±0.1	1.0±0.1	3.2±0.2	1.2±0.3
LDLR	4.2±0.2	1.0±0.1	0.1±0.1	1.1±0.1	2.0±0.1	1.0±0.2	1.7±0.1	1.0±0.2
SREBP1	0.9±0.1	0.9±0.2	2.2±0.2	1.2±01	3.1±0.2	1.1±0.2	3.3±0.1	0.9±0.2
SCD1	1.6±0.2	0.9±0.2	1.1±0.2	1.2±0.3	1.9±0.2	1.1±0.1	1.6±0.2	0.9±0.1
NOR1	4.6±0.1	0.3±0.2	2.2±0.3	0.9±0.2	1.6±0.2	1.0±0.1	1.9±0.2	0.7±0.3
Nur77	6.8±0.2	0.4±0.1	1.4±0.1	0.8±0.2	1.0±0.3	0.9±0.2	0.9±0.3	0.6±0.2
LC3A	2.4±0.2	0.9±0.2	1.3±0.1	1.0±0.3	1.1±0.1	1.1±0.2	1.0±0.3	1.0±0.2
LC3B	1.7±0.1	1.0±0.2	1.1±0.2	1.0±0.1	1.1±0.1	1.1±0.3	1.3±0.1	1.2±0.3

	SKMEL-28							
	DDA		22(R)HC		GW		TO	
	siCT	siLXRβ	siCT	siLXRβ	siCT	siLXRβ	siCT	siLXRβ
ABCA1	0.4±0.1	0.9±0.1	6.5±0.1	1.5±0.1	15.1±2	2.2±0.3	4.2±1.2	2.2±0.3
ABCG1	1.0±0.1	0.8±0.2	2.1±0.2	0.2±0.1	3.2±0.5	1.2±0.1	2.8±0.5	1.1±0.2
ABCG5	3.9±0.4	1.1±0.2	4.5±0.1	1.7±0.3	3.1±0.5	1.1±0.1	3.1±0.5	1.1±0.1
ABCG8	0.2±0.1	0.7±0.2	0.4±0.1	0.7±0.2	1.5±0.1	0.8±0.1	1.5±0.1	0.8±0.1
SULT2B1b	2.9±0.1	1.1±0.2	1.9±0.1	1.0±0.2	2.8±0.1	1.1±0.2	1.8±0.2	1.3±0.2
LDLR	3.2±0.3	1.8±0.2	0.4±0.1	1.1±0.1	1.0±0.2	0.9±0.1	1.0±0.2	0.9±0.1
SREBP1	0.9±0.2	1.0±0.3	2.3±0.2	0.9±0.3	2.6±03	1.5±0.2	1.8±0.3	1.3±0.2
SCD1	2.2±0.2	1.2±0.1	1.0±0.2	0.8±0.1	1.6±0.1	1.1±0.1	1.8±0.2	1.2±0.2
NOR1	4.8±0.2	1.0±0.1	1.8±0.1	1.2±0.2	1.5±0.1	0.8±0.1	1.7±0.1	1.0±0.2
Nur77	8.2±0.2	1.2±0.2	1.6±0.2	0.8±0.2	0.7±0.2	0.9±0.2	1.1±0.2	1.1±0.2
LC3A	1.7±0.2	0.8±02	1.0±0.2	1.0±0.2	0.8±0.2	1.0±0.3	0.8±0.1	0.9±0.1
LC3B	1.6±0.1	0.9±0.3	0.9±0.2	1.0±0.1	0.9±0.1	1.1±0.3	0.9±0.2	0.9±0.1

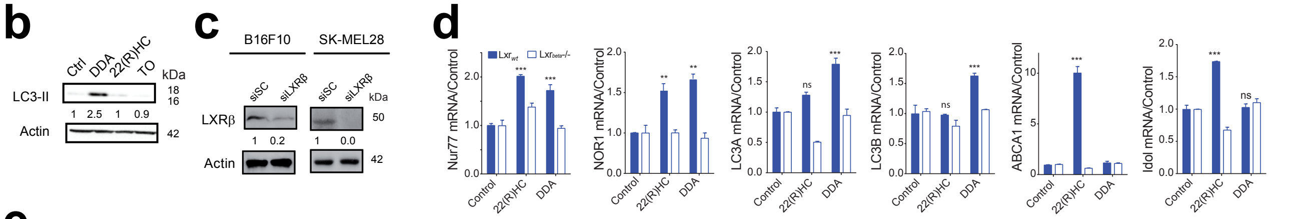

e

Cells	LXR ligand			Sterol amount/total sterol	
	22(R)HC	GW	TO	zymostenol	8-DHC
B16F10	-	-	-	0.13± 0.09	0.05± 0.004
	+	-	-	0.24± 0.08	0.08± 0.006
	-	+	-	0.12± 0.09	0.04± 0.005
	-	-	+	0.11± 0.08	0.05± 0.005
SKMEL-28	-	-	-	0.39± 0.09	0.05± 0.01
	+	-	-	0.37± 0.07	0.05± 0.03
	-	+	-	0.36± 0.08	0.04± 0.02
	-	-	+	0.29± 0.09	0.07± 0.01

f

Cells	DDA	Sterol amount/total sterol				
		zymostenol	8-DHC	lathosterol	7-DHC	desmosterol
shCTRL	-	0.14± 0.02	0.06± 0.02	0.09± 0.03	0.04± 0.006	0.006± 0.002
	+	2.60±0.07	5.81± 0.89	0.41± 0.03	0.03± 0.006	0.01± 0.008
Sh3LXRβ	-	0.17± 0.005	0.03± 0.01	0.28± 0.09	0.05± 0.009	0.03± 0.01
	+	5.78± 0.82	1.52± 0.10	0.71± 0.02	0.03± 0.02	0.01± 0.009
Sh4LXRβ	-	0.52± 0.02	0.07± 0.03	0.27± 0.09	0.05± 0.007	0.07± 0.02
	+	6.57± 0.92	4.32± 0.66	0.90± 0,07	0.39± 0.09	0.02± 0.005

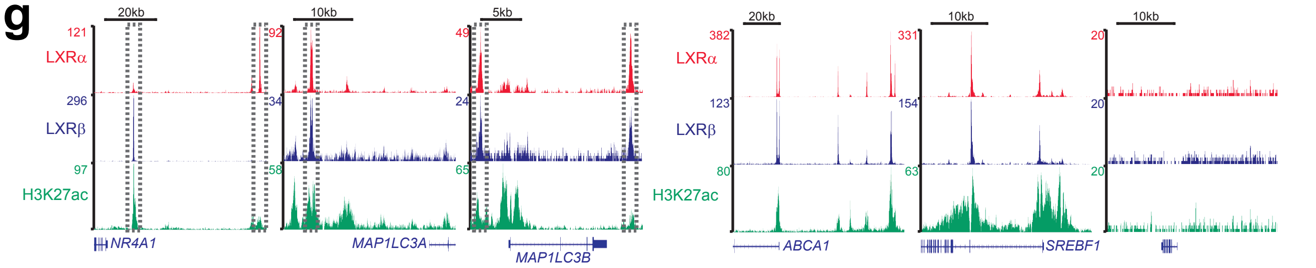

h

GO term Autophagy	Distance LXRBS-TSS (kb)
ATG5	9.3
BNIP1	0.1
CHMP2A	0.5
CHMP3	0.3
CHMP4B	1
CTSD	1.4
FOXO1	6
HMGB1	1.6
ITGB4	2.1
LRSAM1	0.1
MAP1LC3B	3.6
PARK7	0.2
PHF23	3.5
SIRT2	5.7

GO term Autophagy	Distance LXRBS-TSS (kb)
SQSTM1	1.7
STAM	0.3
TBC1D17	0.2
TFEB	5.7
TM9SF1	6.8
TSG101	0.15
UBQLN4	1
VMP1	0.05
VPS28	0.01
VPS37B	7.6
VPS37C	1
VPS39	0.05
XBP1	0.1

GO term Lysosome organization	Distance LXRBS-TSS (kb)
TPP1	7
ABCA1	0.8
HEXB	1.3
ACP2	0.1
LAMTOR1	9
AKTIP	8.2
TFEB	5.7
FAM160A2	4.5

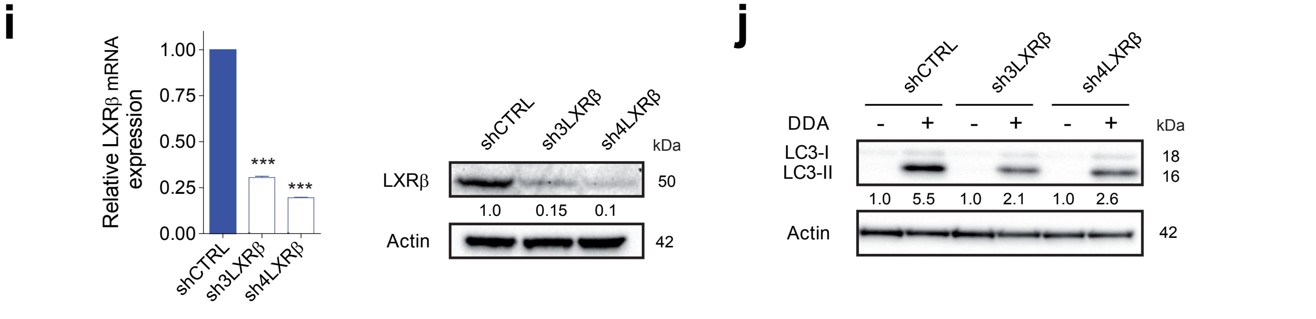

Supplementary Fig. 4. a Regulation of LXR-responsive genes by DDA and other known LXR ligands in B16F10 and SKMEL-28 cells transfected with siRNA targeting LXRβ (siLXRβ) or with control siRNA scramble (siSC). Cells were treated for 8 h with either solvent vehicle, 2.5 µM DDA, 10 µM 22(R) hydroxycholesterol (22(R)HC), 1 µM GW 3965 (GW) or 100 nM TO901317 (TO). Data are expressed as fold change relative to control (solvent vehicle). **b** Effect of LXR ligands on the induction of LC3-II in SKMEL-28 cells. Cells were treated for 24 h with 2.5 µM DDA, 10 µM 22(R)HC, 100 nM TO or solvent vehicle and analyzed for LC3 expression by immunoblotting. **c** Representative immunoblot analysis of LXRβ expression in melanoma cells transfected with siRNA against LXRβ (siLXRβ) or control scramble siRNA (siSC). **d** Real-time PCR analysis of gene expression in mouse embryonic fibroblasts (MEF) cultured with 2.5 µM DDA for 5 h. MEF were from normal (LXR_{wt}) and KO $LXR_{\beta-/-}$ mice. **e** Quantification of sterols accumulation in SKMEL-28 cells either expressing LXRβ (shCTL) or with LXRβ (sh3 or sh4LXRβ) knocked down after 24 h treatment with 2.5 µM DDA or solvent vehicle. Analyses were performed by GC/MS. **f** Quantification of sterols accumulated in melanoma cells after 24 h treatment with 10 µM 22(R)HC, 1 µM GW, 100 nM TO or solvent vehicle. Analyses were performed by GC/MS. **g** Genomic profiles of LXRα, LXRβ and H3K27ac in the regions surrounding the *ABCA1*, *SREBF1*, *ACTA1*, *NR4A1*, *MAP1LC3A* and *MAP1LC3B* genes in HT29 colon cancer cells treated with GW3965, dataset GEO: GSE77039. **h** Computational prediction of putative LXRβ-target genes based on the proximity between their Transcription Start Site (TSS) and LXRβ binding sites (LXRBS). We performed a gene ontology (GO) study using « Autophagy » and « Lysosome organization » terms on these putative LXRβ target genes. **i** Real time PCR and immunoblot of LXRβ expression in SKMEL-28 cells stably transfected with control shRNA (clone shCTRL) or with shRNA against LXRβ (clones sh3LXRβ and sh4LXRβ). Bars represent S.E.M. of 3 experiments performed in triplicate, ***$P<0.001$, *t* test. **j** Immunoblots of LC3 protein expression in cells transfected with shCTRL, sh3LXRβ or sh4LXRβ and treated with 2.5 µM DDA for 24 h. Data from **b** and **i** are the means ± S.E.M. of 3 independent experiments performed in triplicate. (*$P<0.05$, **$P<0.01$, ***$P<0.001$, *t* test).

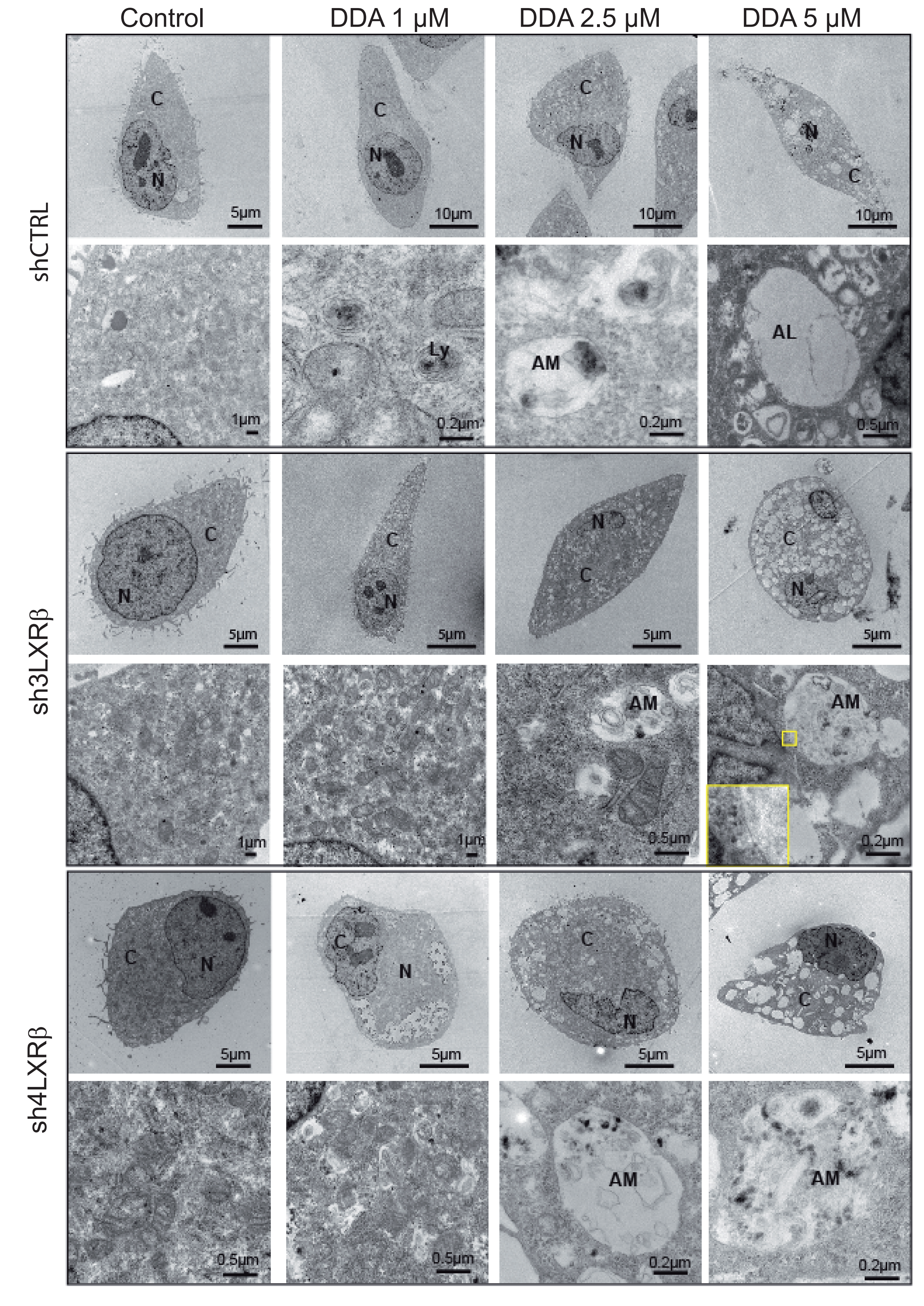

Control
DDA 1 µM
DDA 2.5 µM
DDA 5 µM
shCTRL
sh3LXRβ
sh4LXRβ
C
N
5µm
10µm
1µm
0.2µm
0.5µm
Ly
AM
AL

Supplementary Fig. 5. Involvement of LXRβ in DDA-induced vacuolation of SKMEL-28 cells. SKMEL-28 shCTRL, sh3LXRβ and sh4LXRβ cells were treated for 24 h with solvent vehicle (control) or DDA at the indicated concentrations and ultrastructure modifications were observed by TEM. N: nucleus, C: cytoplasm, Ly: lysosomes, AM: amphisome, AL: autolysosome. Images are representative of 3 independent experiments.

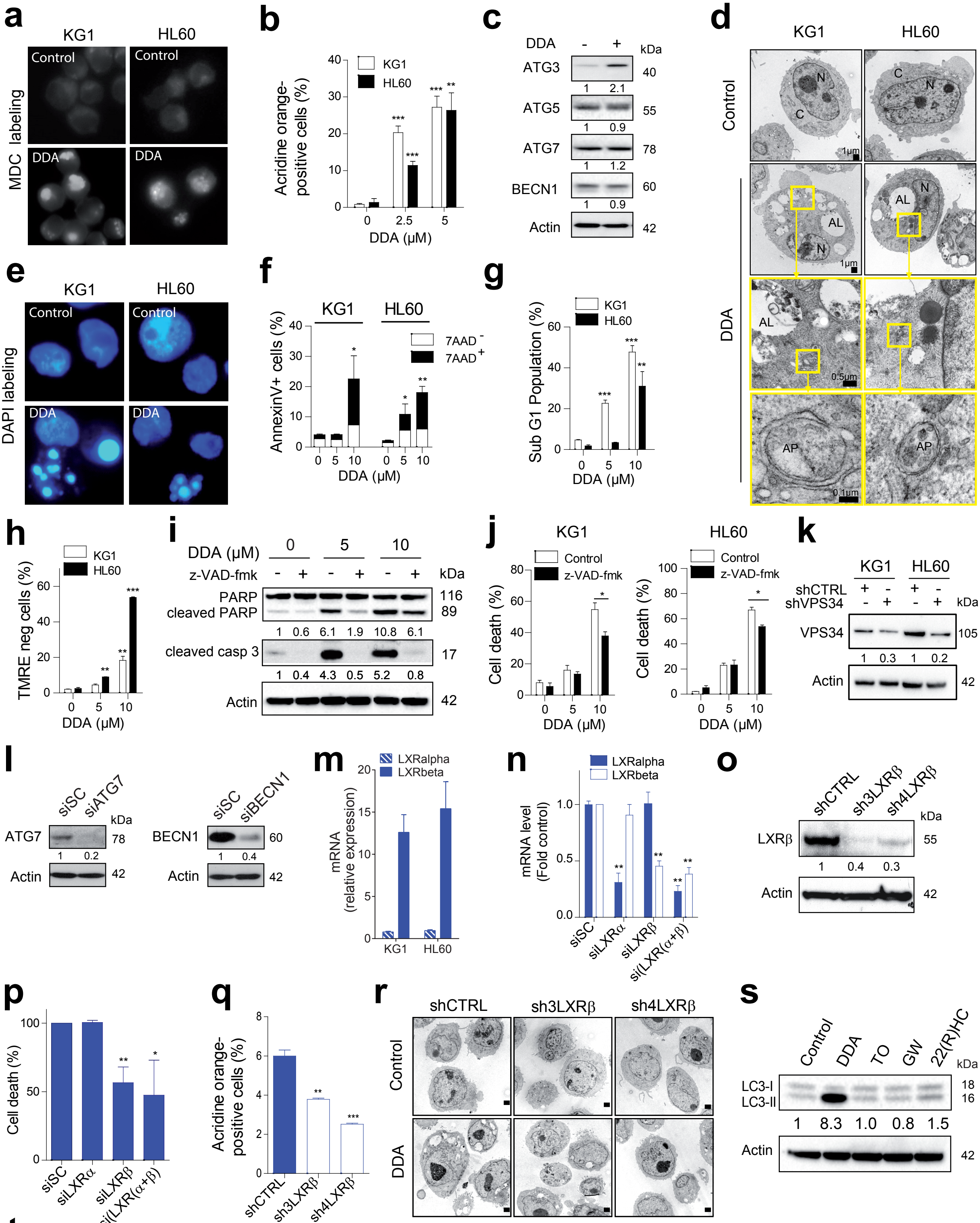

t

	KG1 mRNA levels (Fold over control)				HL60 mRNA levels (Fold over control)			
	TO	GW	22(R)HC	DDA	TO	GW	22(R)HC	DDA
Nur77	1.5	0.8	1.1	8.8	0.4	1.3	0.8	11.2
NOR1	1.1	1.8	6.3	12.2	2.7	0.3	0.7	6.3

Supplementary Fig. 6. DDA induced the appearance of autophagic vesicles in AML cells (KG1 and HL60). **a** Representative images of cells treated with 5 μM DDA for 24 h and then stained with MDC. **b** Flow cytometry analysis of KG1 and HL60 cells exposed to DDA and then stained with acridine orange. Data represent the percentage of acridine orange-positive cells and the mean of 3 independent experiments. Bars represent S.E.M. (*P<0.05, **P<0.01, ***P<0.001, *t* test). **c** Immunoblot analysis of ATG3, ATG5, ATG7 and Beclin-1 (BECN1) in KG1 cells treated with solvent vehicle or 5 μM DDA for 24 h. **d** TEM images of KG1 and HL60 cells treated with solvent vehicle or 5 μM DDA for 24 h. N: nucleus, C: cytosol, AP: autophagosomes, AL: autolysosomes. **e** Images of KG1 and HL60 cells treated for 24 h with 10 μM DDA or solvent vehicle. Nuclei were stained with DAPI. **f** Apoptosis was assessed by annexin-V/7-AAD staining of KG1 and HL60 cells treated for 24 h with DDA or solvent vehicle. **g** Cell cycle analysis of KG1 and HL60 cells treated with DDA for 24 h. **h** DDA induced a reduction in the mitochondrial membrane potential ($\Delta\psi m$) in KG1 and HL60 cells. AML cells were treated with DDA for 24 h, then stained with TMRE (tetramethyl rhodamine ethyl ester) and analyzed by flow cytometry. **i** Immunoblot analysis of caspase-3 and PARP expression in AML cells treated with DDA for 24 h in the absence or presence of 50 μM z-VAD-fmk (a caspase inhibitor). **j** Effect of z-VAD-fmk on DDA-induced cytotoxicity in KG1 and HL60 cells. Cells were incubated for 24 h with increasing concentrations of DDA in the absence or presence of z-VAD-fmk (50 μM). **k** Immunoblot analysis of VPS34 expression in KG1 and HL60 cells stably transfected with control ShRNA (shSC) or shRNA against VPS34 (shVPS34). **l** Immunoblot analysis of ATG7 and BECN1 expression in KG1 cells transfected with scrambled (siSC), ATG7 (siAtg7) or BECN1 (siBECN1) siRNA for 24 h. **m** qRT-PCR analysis of LXRα and LXRβ expression in HL60 and KG1 cells. (n) qRT-PCR analysis of the expression of LXRα and LXRβ in KG1 cells transiently transfected with scrambled (siSC) or siRNA against LXRα (siLXRα), LXRβ (siLXRβ) or both LXRα and LXRβ (siLXRα + β). **o** Immunoblot of LXRβ expression in KG1 cells stably transfected with control shRNA (clone shCTRL) or with shRNA against LXRβ (clones sh3LXRβ and sh4LXRβ). **p** Cell death was measured by trypan blue exclusion in KG1 cells transiently transfected with scrambled siRNA (siSC) or siRNA against LXRα (siLXRα), LXRβ (siLXRβ) or both LXRα and LXRβ (siLXRα + β) and then treated for 24 h with solvent vehicle or 5 μM DDA. DDA-induced cell death is expressed as the percentage relative to control (vehicle)-treated cells. **q** Flow cytometry analysis of cells permanently transfected with shCTRL, sh3LXRβ and sh4LXRβ and then treated for 24 h with 5 μM DDA and stained with acridine orange. **r** Representative images of cells permanently transfected with shCTRL, sh3LXRβ and sh4LXRβ and treated for 24 h with 5 μM DDA and analyzed by TEM. **s** Immunoblots of LC3 protein expression in KG1 cells treated with 5 μM DDA, 2 μM T0, 2 μM GW or 10 μM 22(R)HC for 24 h. **t** qRT-PCR analysis of the expression of Nur77 and NOR1 in KG1 and HL60 cells treated for 24 h with either solvent vehicle, 5 μM DDA, 2 μM T0, 2 μM GW or 10 μM 22R-HC. Data are expressed relative to the control (solvent vehicle). Data from b, f, g, h, j, p, q are the means ± S.E.M. of 3 independent experiments performed in triplicate (*P<0.05, **P<0.01, ***P<0.001, *t* test). Images are representative of 3 independent experiments.

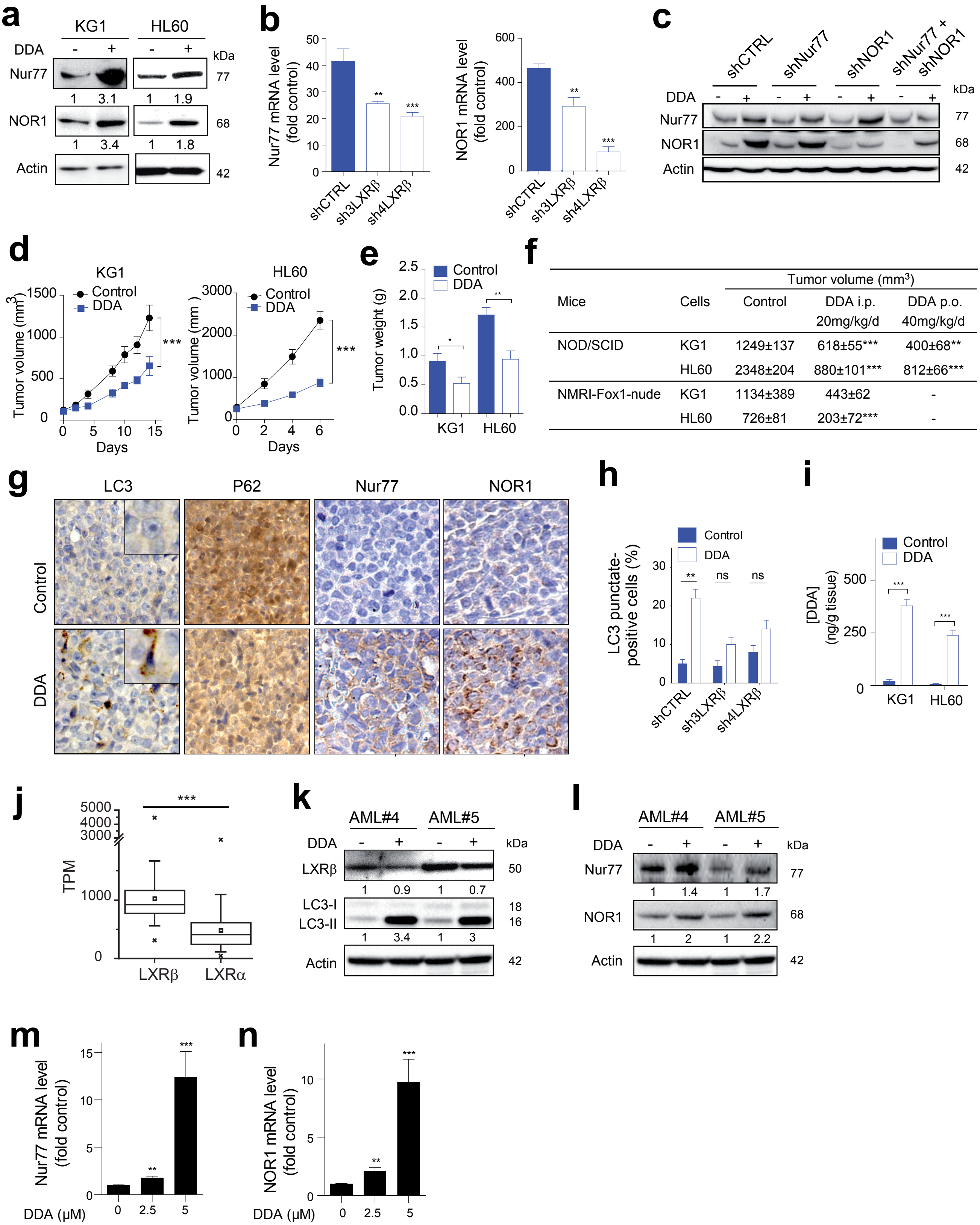

f

Mice	Cells	Tumor volume (mm³) Control	DDA i.p. 20mg/kg/d	DDA p.o. 40mg/kg/d
NOD/SCID	KG1	1249±137	618±55***	400±68**
	HL60	2348±204	880±101***	812±66***
NMRI-Fox1-nude	KG1	1134±389	443±62	-
	HL60	726±81	203±72***	-

Supplementary Fig. 7. a Immunoblots of Nur77 and NOR1 proteins in cells treated or not for 24 h with 5 µM DDA. **b** Real-time PCR of Nur77 and NOR1 expression in KG1 cells stably transfected with control shRNA (clone shCTRL) or with shRNA against LXRβ (clones sh3LXRβ and sh4LXRβ) and treated with 10 µM DDA for 24 h. **c** Immunoblots of Nur77 and NOR1 protein expression in KG1 cells stably transfected with control shRNA (clone shCTRL), shRNA against Nur77 (shNur77), shRNA against NOR1 (shNOR1), or shRNAs against both Nur77 and NOR1 (shNur77 + shNOR1). **d** NOD/SCID mice (n=16-20 mice/group) engrafted with KG1 cells or HL60 cells were treated with DDA (20 mg/kg/day, i.p.) or solvent vehicle (control). Tumor growth was monitored over time and was analyzed for significance using repeated measures ANOVA (***P<0.001). The mean tumor volumes ± SEM are shown. **e** Average tumor weight at the end of the experiments in **d**. *P<0.5, **P<0.01, ANOVA. **f** Mean tumor volumes measured at the end of the experiments for HL60 and KG1 grafted into NOD/SCID or NMRI-Fox1-nude mice treated with DDA (40 mg/kg/day, p.o. or 20 mg/kg/day, i.p.) or solvent vehicle (control) (n=10-20 mice per group). Values represent the means ± S.E.M. (**P<0.01, ***P<0.001, ANOVA). **g** Histochemical analysis of the expression of LC3 and P62 in HL60 tumor sections isolated from NOD/SCID mice treated or not with DDA (20 mg/kg/day, i.p.). **h** Quantification of LC3 punctate cells in shCTRL and shLXRβ KG1 tumors. **i** Quantification of DDA levels in tumors 4 h after the final administration of DDA. **j** Box plot of TCGA RNA-seq data from patients with melanoma showing that LXRβ is the predominant LXR isoform expressed. ***P<0.001. **k**, **l** Immunoblotting of the indicated proteins in primary AML cells exposed to vehicle or 5 µM DDA for 24 h. **m**, **n** qPCR analysis of the expression of Nur77 and NOR1 in primary AML cells from 10 patients exposed to vehicle or DDA for 24 h. Bars represent S.E.M. (**P<0.01, ***P<0.001, *t* test). Data from b, h, i represent means ± S.E.M. of triplicate samples (>100 cell analyses per sample), **P<0.01, ***P<0.001, *t* test. All data or images are representative of 3 independent experiments.

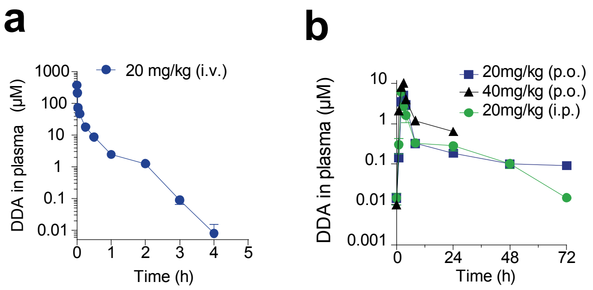

c

Administration R oute	p.o.	i.p.	i.v.
Dose (mg/kg)	20	20	20
Plasma clearance (l/h/kg)	1.57	1.68	1.62
$T_{1/2}$ distribution (h)	-	-	0.1
$T_{1/2}$ elimination (h)	1.0	0.9	0.6
$T_{1/2}$ absorption (h)	43	19	-
Bioavailability (%EP)	103	96	100
C_{max} (µM)	5.05	6.10	370
Tmax (h)	3	2	0.002

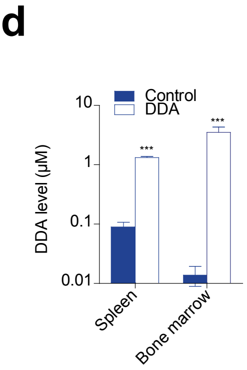

Supplementary Fig. 8. a DDA plasma concentrations were analyzed at different times after the treatment of NSG mice with a single administration of DDA at 20 mg/kg (i.v.). **b** DDA plasma concentrations were analyzed at different times after the treatment of NSG mice with a single administration of DDA at 20 or 40 mg/kg (p.o.) or 20 mg/kg (i.p.). **c** Summary of pharmacokinetic parameters of DDA injected into NSG mice. **d** DDA concentrations were measured in the spleen and bone marrow after daily i.p. administration of DDA at 20 mg/kg for 21 days. Bars represent S.E.M. (***P<0.001, **P<0.01, *t* test).

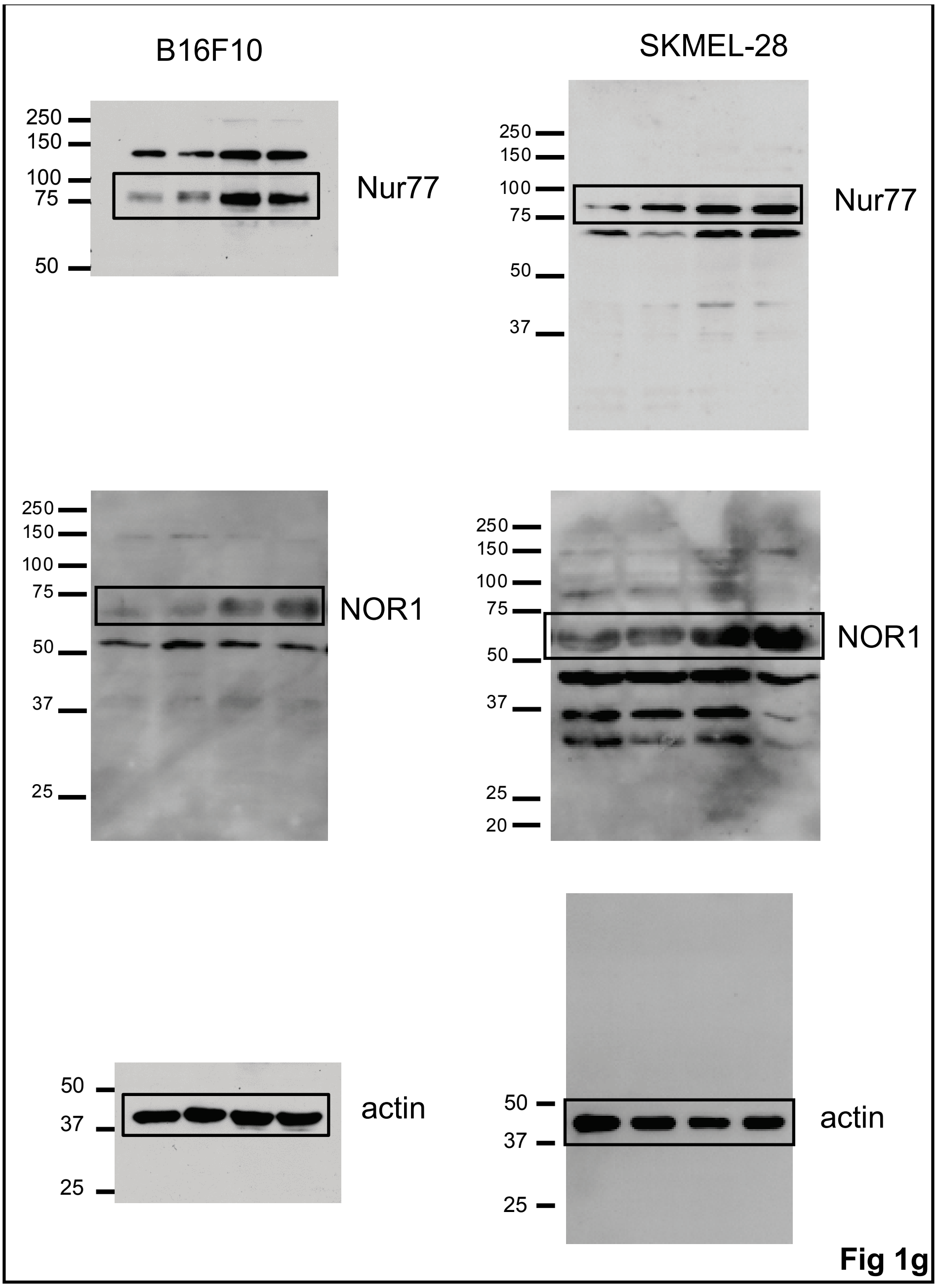

Supplementary Figure 9. Original western blot images

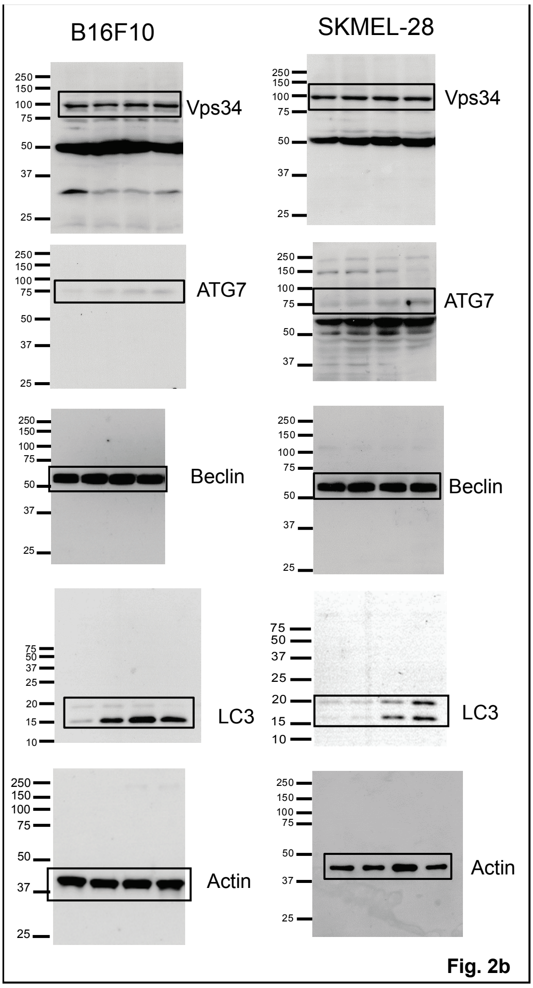

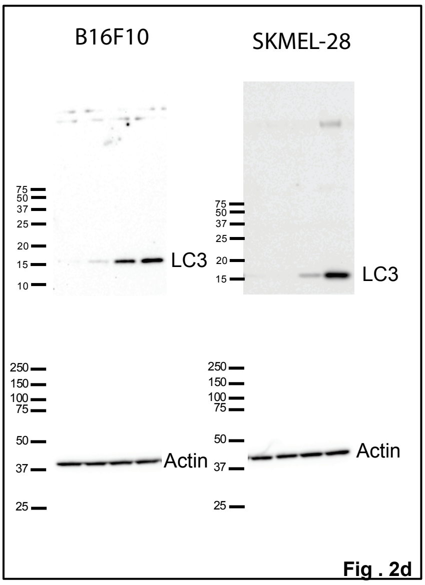

Supplementary Figure 9. continued

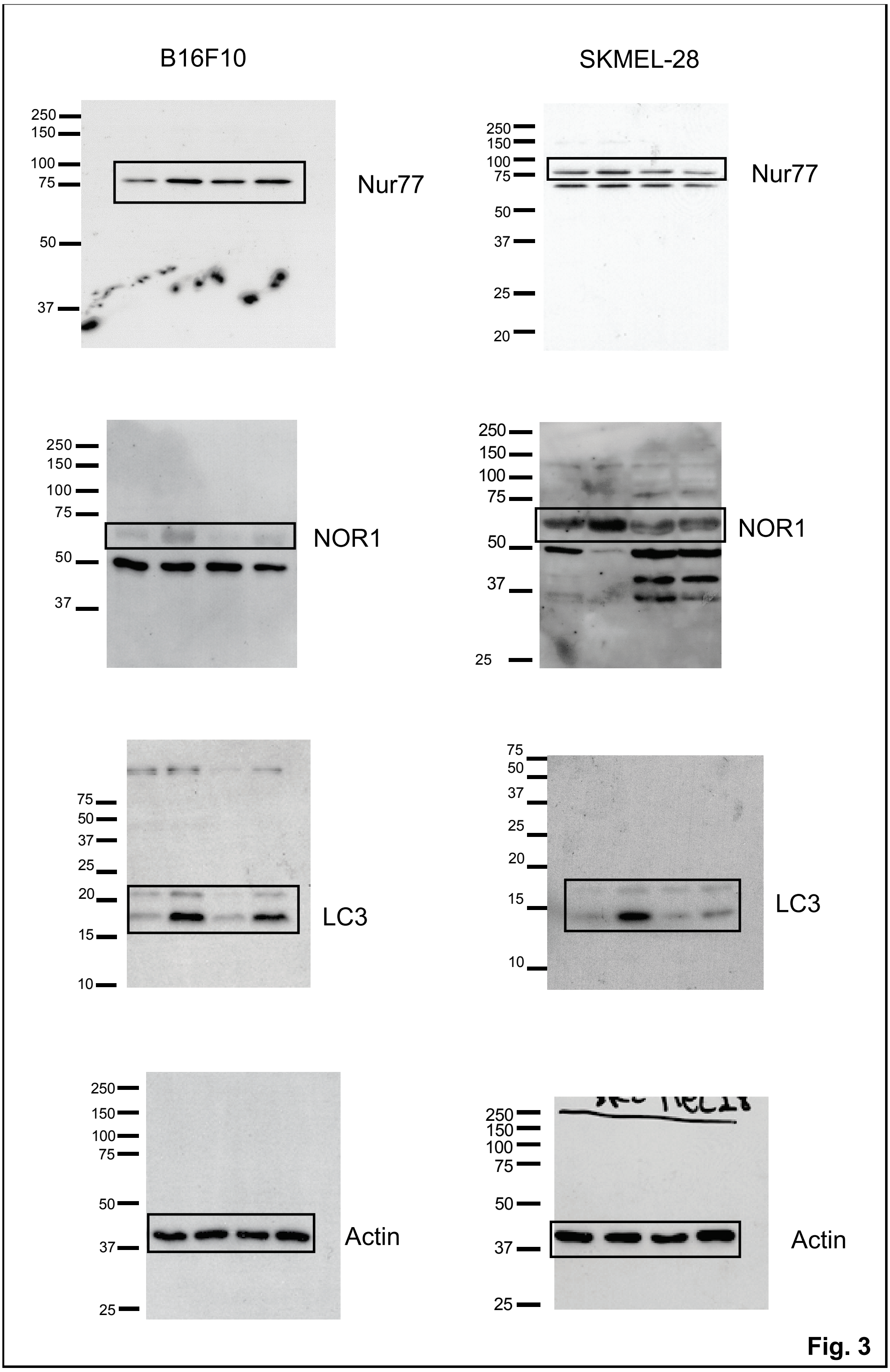

Supplementary Figure 9. continued

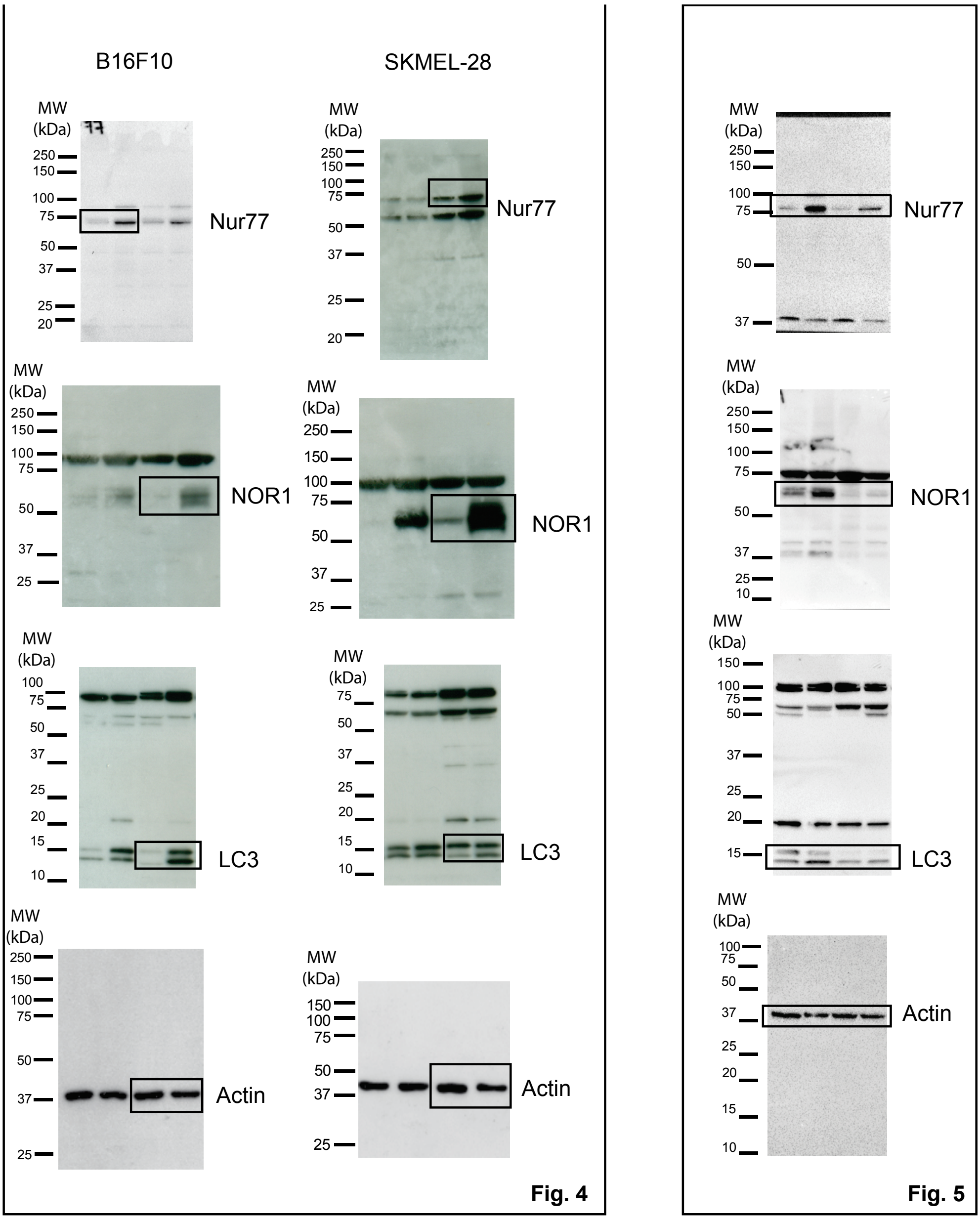

Supplementary Figure 9. Continued

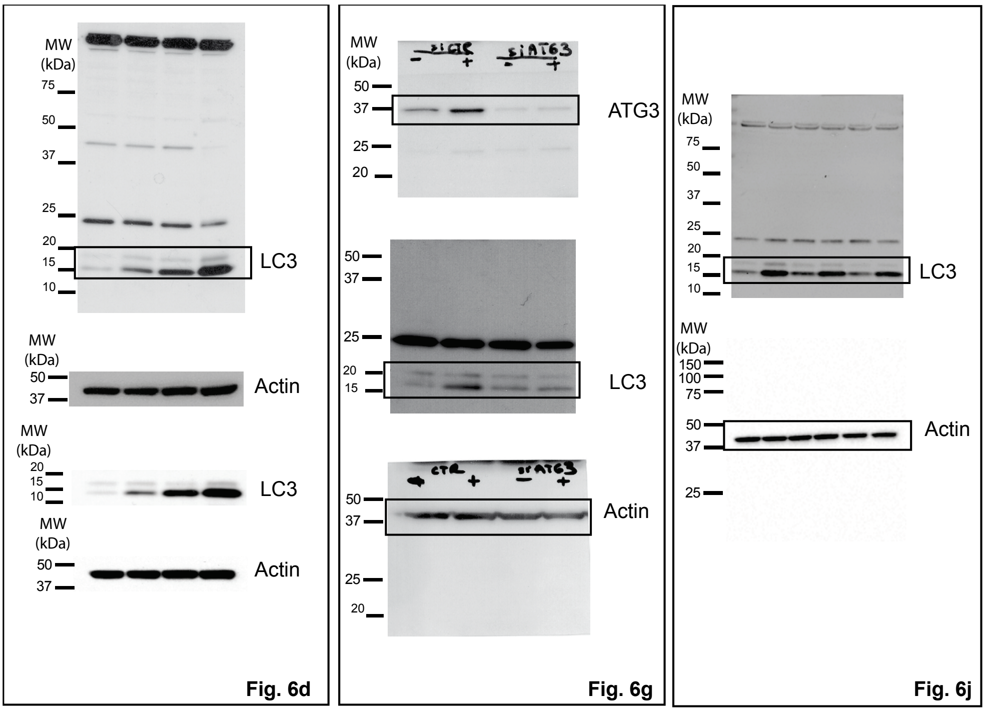

Supplementary Figure 9. continued

Human primers	Forward	Reverse
LXRα	CTT GCT CAT TGC TAT CAG CAT CTT	ACA TAT GTG TGC TGC AGC CTC T
LXRβ	GCT AAC AGC GGC TCA AGA ACT AA	GCT CCG TGA AGT GGG CAA A
ABCA1	ATG AGG ACA ACA ACT ACA AAG CC	GGG AAA GAG GAC TAG ACT CCA GA
ABCG1	TTT GAG GGA TTT GGG TCT AGA AC	CCC CTT TAA TCG TTT TGT CTG CT
ABCG5	TGG GAC ATC ACA TCT TGC CG	GAA GCC AAG CAT CTC CTC TG
ABCG8	CCA CTC CCC AGG ATA CCT CG	TCA AAC CAA GGG ACC TGA GAG
SULT2B1	CGG GAC GAC GAC ATC TTT AT	CAC CCA CAA TAG GTC TCA CAC
LDLR	GGA CCA ACG AAT AGC TTA GGA CA	CTA GGC ACT TAG TAG CCA CCC
SREBP-F1	CAG CCC CAC TTC ATC AAG G	ACT AGT TAG CCA AGA TAG GTT CCG
SCD1	ACC GCT CTT ACA AAG CTC GG	CCA CGT CGG GAA TTA TAG AGG AT
NOR1	TCC ATC AGG TCA AAC ACT GC	GGA AAA GTC TTT AAT AGA GTC G
NUR77	ATG GTG AAG GAA GTT GTC CGA AC	CCG GAG AGC AGG TCG TAG AAC
LC3A	CGT CCT GGA CAA GAC CAA GT	CTC GTC TTT CTC CTG CTC GT
LC3B	AAG GCG CTT ACA GCT CAA TG	CTG GGA GGC ATA GAC CAT GT
TFEB	CAA GGC CAA TGA CCT GGA C	AGC TCC CTG GAC TTT TGC AG
cylophilin A	GCA TAC GGG TCC TAG GCA TCT TAG TC	ATA GGT AGA TCT TCT TAG CTA GGT CTT AGC
GAPDH	CAT CGT GGA AGG GCT CAT GAC	CTT GGC AGC ACC AGT GGA TG
cyclophilin B	GAT GGC ACA GGA GGA AAG AG	AAC TTT GCC GAA AAC CAC AT
β-actin	CAG CTT CTT TGC AGC TCC TT	GCA GCG ATA TCG TCA TCC A
TBP	AC GAA CCA CGG CAC TGA TT	TTT TCT TGC TGC TGC CAG TCT GGA C
L32	GTGCCGAGATCGCTCACA AT	CAGCTCTTTCCACGATGGC

Supplementary Table 1: List of human primers.
Primer sequences used for RT-qPCR analysis in human samples.

Mouse primers	Forward	Reverse
LXRα	GTG GAA GAC AGA ACC TCA AGA TG	GAG CAG CGT CCA TCC AGA G
LXRβ	GGA GGT GTA AGT GAG GCT GAG	AAC CTG CCA GAT GGA TGC CTT C
ABCA1	GGA-GCT-GGG-AAG-TCA-ACA-AC	ACA-TGC-TCT-CTT-CCC-GTC-AG
ABCG1	GTG CTG TTC GCT GCT CTG	GGC TGG GAT GGT GTC AAA G
ABCG5	TCA GGA CCC CAA GGT CAT GAT	CCG TTC ACA TAC ACC TCC CC
ABCG8	GAC AGC TTC ACA GCC CAC AA	GCC TGA AGA TGT CAG AGC GA
SULT2B1	GGG ACG ACG ACA TCT TCA TT	ATT GAA GGC GCT TAT GAT GG
LDLR	TGA CTC AGA CGA ACA AGG CTG	ATC TAG GCA ATC TCG GTC TCC
SREBP-1c	AAG GCC ATC GAC TAC ATC CG	GCT TTT GTG TGC ACT TCG TAG G
SCD1	CTG TAC GGG ATC ATA CTG GTT C	GCC GTG CCT TGT AAG TTC TG
NOR1	AGG ATT CAC TGA TCT CCC CAA	GAT GCA GGA CAA GTC CAT TGC
NUR77	ATG CCT CCC CTA CCA ATC TTC	CAC CAG TTC CTG GAA CTT GGA
LC3A	CTA TGA ACA GGA GAA GGA TGA AG	ACT CAG AAG CCG AAG GTT
LC3B	TGT GTA ACT GTC TCT GTA AG	TCT TCT GTT GCT GTT GTC
IDOl	AGC-GGC-CTC-TAC-CGA-GCC-AT	CGC-CAA-GTG-GCC-CTT-CAG-GT
Cylophilin A	TAT AAG GGT TCC TCC TTT CAC AGA A	GGA CCT GTA TGC TTT AGG ATG AAG
β-actin	AGC CAT GTA CGT AGC CAT CCA	TCT CCG GAG TCC ATC ACA ATG
GAPDH	GTG TTC CTA CCC CCA ATG TGT	TTG TCA TAC CAG GAA ATG AGC TT
TBP	TCC CCC TCT GCA CTG AAA TC	AGT GCC GCC CAA GTA GCA

Supplementary Table 2: List of mouse primers
Primer sequences used for RT-qPCR analysis in mouse samples

**ChIP primers**	**Forward**	**Reverse**
ABCA1	ATT GCG AGC GAG AGT GAG TG	GAG AAC CGG CTC TGT TGG TG
SREBP-F1	ACT GAC ATC CAC CGA ATG CC	TGT GTT CCC TAA AAG AGG GGG
NR4A1	CGG AAG TGA GTT GTA CGG GC	AAG ATC CCC CTC ATG TGG AC
MAP1LC3A	TGC CAA ATA TGT GAG TCA TGG A	CCC ACC AGG CAC TAT TAT CCC
MAP1LC3B	TGG AGC AAA TTC TCC GCG TG	ATG TGA CCG ACC ACG TGA C
SCD1	GTT AAA CTC TGG GAC GCA GGA	ATG TGA CCG ACC ACG TGA C
LDLR	ACG ATA TTG TCA AAG CGG GGT	GTC GAG CTT TGG AAT CTG CTG
NR4A3	ATT GAC GTC TCG CAT TCC AGG	CTC GGC ACG TCA TTT ATG CCA
TFEB	TCT AGC CTG GTC CTG GAG TC	ACC CAC TGC CTG TCT CAC TA

Supplementary Table 3: List of ChIP primers.
Primer sequences used for the ChIP assays.

Antibody	Dilution	Manufacturer	Reference
Caspase-3 (WB)	1/1000	Cell Signaling	9662
Beclin (D40C5) (WB)	1/1000	Cell Signaling	3495
Atg7 (D12B11) (WB)	1/1000	Cell Signaling	8558
Atg5 (D1G9) (WB)	1/1000	Cell Signaling	8540
Atg3 (WB)	1/1000	Cell Signaling	3415
LC3 (WB)	1/5000	Sigma-Aldrich	L8918
LC3 (5F10) (IHC)	1/100	NanoTools	0231-100/LC3-5F10
Bax (WB)	1/1000	Millipore	06-499
Bcl-2 (WB)	1/500	Millipore	04-436
Lamp1 (WB and IF)	1/500	Abcam	ab24170
LXRα (WB)	1/200	SantaCruz	sc-1202
LXRβ (WB)	1/200	SantaCruz	SC-34341
LXRβ (Chip)	4 µg/test	Active Motif	61177
Nur77 (WB)	1/500	Active motif	40982
Nur 77 (IHC)	1/50	LS-Bio	LS-B2456
NOR1 (WB)	1/1000	R&D systems	PP-H7833-00
NOR1 (IHC)	1/200	LS-Bio	LS-A2341
PARP (46D11) (WB)	1/1000	Cell Signaling	9532
Vps34 (WB)	1/1000	Cell Signaling	3358
Actin (WB)	1/10 000	Millipore	MAB1501
CD34-PeCy7 (8G12) (FC)	20 µl/test	BD Biosciences	348057
CD38-APC (clone HB7) (FC)	5 µl/test	BD Biosciences	340439
CD123-PE (clone 9F5) (FC)	20 µl/test	BD Biosciences	551065
CD45-V450 (clone H130) (FC)	5 µl/test	BD Biosciences	564915
P62 (IHC)	1/100	Progen	GP-62C

Supplementary Table 4: list of antibodies
Antibodies used for western blot (WB), immunohistochemistry (IHC), immunofluorescence (IF), flow cytometry (FC) or Chromatin immunoprecipitation (ChIP) analysis as indicated.
