# Supplementary material for: Dendrogenin A drives LXR to trigger lethal autophagy in cancers
Source: Nat Commun. 2017 Dec 4;8:1903. doi: 10.1038/s41467-017-01948-9 (PMC5712521; doi:10.1038/s41467-017-01948-9)
Supplement: Supplementary file 3 — Description of Additional Supplementary Files [file 41467_2017_1948_MOESM3_ESM.pdf]

**Description of Additional Supplementary Files**

File Name: Supplementary Data 1
Description: Clinical characteristics of primary AML patients.
